# Supplementary material for: ARIAS: An AR-based interactive advertising system
Source: PLoS One. 2023 Sep 29;18(9):e0285838. doi: 10.1371/journal.pone.0285838 (PMC10540975; doi:10.1371/journal.pone.0285838)
Supplement: Attachment — Submitted filename: Response to Reviewers.docx [file pone.0285838.s001.docx]

Please Note: The bold **LineXXX** in the following responses refers to the line in the ‘Manuscript’, not ‘Revised Manuscript with Track Changes’.

| **Role** | **Suggestions or questions** | **Responses** |
| --- | --- | --- |
| Academic Editor | The reviewers have found minor issues in the manuscript. More specifically, the reviewer 1 asks for clarifications of terms used in the manuscript and more discussions regarding the comparison of the proposed system and the state of the art. The reviewer 2 has some concerns regarding the statistical analysis which was found not rigorous and appropriate. Moreover, the reviewer requests to define the acronym ARVS, provide clarifications regarding the use of the system and discuss the learning curve of using the proposed interaction method. | Thank you very much. We have given the corresponding modifications, as detailed below. |
| Reviewer #1 | Abstact Line 1: describe the meaning of ARVS | Thank you very much for all of your helpful suggestions.  We named the system “A video system based on augmented reality” at first, and ARVS was its acronym. Since it has been upload to the web site of GitHub and open with a unique URL, we haven't changed its name later. Now we change it to ARIAS to avoid ambiguity and highlight its interactivity, and explained it in the title and abstract of the manuscript. **(Line2-3, Line11-13)** |
| Reviewer #1 | Authors could describe the Mixed Reality and make a comparison between this system and theirs | We discuss the advantages and disadvantages of AR in this system compared to MR, which with the similar theoretical technology with AR. The Similar suggestions are given by the other reviewer, and we added the 'System features' section for comprehensive descripting  **(Line567-603)** |
| Reviewer #1 | Authors could describe potential applicability of such system and the reason why it is different with the other systems. | This article has four keyword: Advertising, Augmented reality, Gesture and Interactive, we found none effective comparable system in GitHub and Web of Science with any three of them, including the use of their synonyms and acronyms. (Perhaps this system will become the compared object if it can be published). Therefore, this system was compared with the traditional way of advertising. We analysed the potential applicability of such system according to the results. **(Line516-524, Line558-566)** |

| **Role** | **Suggestions or questions** | **Answers** |
| --- | --- | --- |
| Reviewer #2 | The submission titled “ARVS: Design of an interactive advertising system” discusses a methodology to produce a virtual cube, akin to an augmented reality hologram, that shows advertising videos to better grasp customer attention. The authors use established external libraries that identify and transform individual video frames, and then implement hand gestures to incorporate human interactions. A user's fingertips serve as a mouse pointer and hand gestures are used to translate, rotate, and zoom the virtual cube. Overall, this was a well-written manuscript, the data algorithms were adequately explained, and the significance of the work was properly highlighted from a psychological standpoint. I only have a few comments listed below. | Thank you very much for praising our work and for all of your helpful suggestions. Your questions will be answered in detail below. |
| Reviewer #2 | 1. The acronym ARVS should be defined. I assume it stands for augmented reality video system? Is this a novel name that the authors created? | Yes, we named the system “An augmented reality video system” at first, and ARVS was its acronym. Since it has been upload to the web site of GitHub and open with a unique URL, we haven't changed its name, even though it has been added the function of gesture manipulation later. Now we change it to ARIAS to avoid ambiguity and highlight its interactivity, and explained it in the title and abstract of the manuscript. **(Line2-3, Line11-13)** |
| Reviewer #2 | 2. Is there an indication given to the user for which state is currently selected (e.g., translation, rotation, zooming, etc.)? Otherwise, can virtual buttons be displayed to the user to facilitate interactions with the virtual cube? | Thanks again for the helpful idea. We have always felt that these states and instructions are easy to remember from our own point of view, but ignored that it may be a challenge for a customer who has just contacted the system for a few minutes. Now, the virtual buttons and text indication were both given to the user. **(Line418-428,Fig 11)** |
| Reviewer #2 | 3. How steep is the learning curve to use the gesture interactions? If there is a new customer that has not used ARVS, would they become too confused to benefit from the virtual cube advertising? There would be a great benefit to test the system on users that ranges of prior experience with ARVS. | We invited 22 people who did not contacted the system to participate in the test of the system. Each person tested the system 6 times, and then obtained the number of errors and the time taken for each test. We drew the error curve and time taken curve, the results then were discussed according to the curve characteristics. **(Line466-515, Line526-557)** |
| Reviewer #2 | 4. More explanation is required to explain how gestures are tracked. Is there another camera directly across the user? Or could there be a camera placed behind the user? How adaptable is the system to augmented reality headsets that can similarly record hand gestures and/or gaze data? | For the gesture tracking, it work with the help of the library MediaPipe, by which the gesture data is inferred from the image based on deep learning. We just mentioned briefly **(Line297-303)** because the working principle is described in detail in the 31th reference. So there is just only one camera required by this system. **(Line593)**  For the system adaptable to headsets, the similar suggestions are given by the other reviewer, and we added the System features section for comprehensive descripting. **(Line567-603)** |
