# Supplementary material for: ARIAS: An AR-based interactive advertising system
Source: PLoS One. 2023 Sep 29;18(9):e0285838. doi: 10.1371/journal.pone.0285838 (PMC10540975; doi:10.1371/journal.pone.0285838)
Supplement: Attachment — Submitted filename: Response to Reviewers.docx [file pone.0285838.s002.docx]

| **Role** | **Comments** | **Responses** |
| --- | --- | --- |
| Academic Editor | All the issues have been addressed in the revised version of the paper. A final remark is raised by a reviewer that regards a figure in the manuscript. Considering the reviewer's comment I recommend accepting the paper after a minor revision.  (According to the reviewers' comments, the majority of the concerns have been answered/addressed.  A final comment regards the format of Figure 10 that should be fixed.) | Thank you very much for considering accepting our manuscript. The Fig.10 as well as Fig.12-15 has been formatted as the comment of Reviewer #2. |
| Reviewer #1 | The Authors has replied to all comments satisfactorily and for this reason I suggest accepting the MS. | Thank you very much for suggesting accepting our manuscript. |
| Reviewer #2 | The authors have adequately answered/addressed my concerns. My final comment is to appropriately format Fig. 10 with axes labels. | Thank you very much for your kind comment. The Fig.10 as well as Fig.12-15 has been formatted with axes labels. |
